# A Novel Phenolic Compound, Chloroxynil, Improves *Agrobacterium*-Mediated Transient Transformation in *Lotus japonicus*

**DOI:** 10.1371/journal.pone.0131626

**Published:** 2015-07-15

**Authors:** Mitsuhiro Kimura, Sean Cutler, Sachiko Isobe

**Affiliations:** 1 Department of Frontier Research, Kazusa DNA Research Institute, Kisarazu, Chiba, Japan; 2 Department of Botany and Plant Sciences, Center for Plant Cell Biology and Institute for Integrative Genome Biology, University of California Riverside, Riverside, California, United States of America; Centro de Investigación y de Estudios Avanzados del IPN, MEXICO

## Abstract

*Agrobacterium*-mediated transformation is a commonly used method for plant genetic engineering. However, the limitations of *Agrobacterium* host-plant interactions and the complexity of plant tissue culture often make the production of transgenic plants difficult. Transformation efficiency in many legume species, including soybean and the common bean, has been reported to be quite low. To improve the transformation procedure in legumes, we screened for chemicals that increase the transformation efficiency of *Lotus japonicus*, a model legume species. A Chemical library was screened and chemicals that increase in transient transformation efficiency of *L*. *japonicus* accession, Miyakojima MG-20 were identified. The transient transformation efficiency was quantified by reporter activity in which an intron-containing reporter gene produces the GUS protein only when the T-DNA is expressed in the plant nuclei. We identified a phenolic compound, chloroxynil, which increased the genetic transformation of *L*. *japonicus* by *Agrobacterium tumefaciens* strain EHA105. Characterization of the mode of chloroxynil action indicated that it enhanced *Agrobacterium*-mediated transformation through the activation of the *Agrobacterium vir* gene expression, similar to acetosyringone, a phenolic compound known to improve *Agrobacterium*-mediated transformation efficiency. Transient transformation efficiency of *L*. *japonicus* with 5 μM chloroxynil was 60- and 6- fold higher than that of the control and acetosyringone treatment, respectively. In addition, transgenic *L*. *japonicus* lines were successfully generated by 5 μM chloroxynil treatment.Furthermore, we show that chloroxynil improves *L*. *japonicus* transformation by *Agrobacterium* strain GV3101 and rice transformation. Our results demonstrate that chloroxynil significantly improves *Agrobacterium tumefaciens*-mediated transformation efficiency of various agriculturally important crops.

## Introduction

Genetically modified (GM) crops are commonly used for improving agricultural production and environmental protection. The first GM crop, FLAVR SAVR tomato, was commercially introduced in 1994 [1]. At present, the global cultivated area of GM crops is 170.3 million hectares in 28 countries [2], where GM soybean and maize comprise 72% and 28% respectively, of the total cultivated area [3]. These results indicate that the cultivation of GM crops is widely increasing across the world. However, current GM techniques are capable for only limited plant species and ecotypes, and do not always work for crop cultivars commonly used in agriculture. Therefore, the establishment and improvement of common GM techniques that cover a wider range of plant species is greatly needed.

Transgenic plants are produced by a two-step process, transgene delivery and plant regeneration. Several methods have been developed for transgene delivery into plant genomes, such as *Agrobacterium*-mediated transformation, direct gene transfer into protoplasts and particle bombardment. *Agrobacterium*-mediated transformation is the most commonly used method because of its higher transformation frequency, integration of long DNA segments and stable expression of the transgene due to the low copy number of T-DNA insertions [4]. In addition, an *in planta* transformation method which enables transformation of plants without the need for plant tissue culture, has allowed many plant researchers to adopt functional genomics and molecular breeding approaches to analyze the function of their gene of interest [5–7].

*Agrobacterium* delivers genes located between left border (LB) and right border (RB) of the Ti plasmid into plant genomes via the T-DNA transport machinery in which *Agrobacterium* Vir proteins and host proteins are involved. Thus, efficiency of transgene delivery is affected by several factors, such as: explant sources, tissue culture medium, *Agrobacterium* strain, binary vector types, host plant species and co-cultivation conditions. To optimize and increase the success rate of transgene delivery, a combination of multiple factors needs to be examined. The addition of novel chemicals into the plant tissue culture media is considered to be a simple approach for improving transformation efficiency because it does not required any major modification to the basic standard protocol. For example, the *vir* genes, which regulate T-DNA transport, are activated by phenolic compounds exuded from wounded sites of the plant tissue [8]. Therefore, several studies reported that the host range of *Agrobacterium*-mediated transformation has been expanded by the addition of a phenolic compound, such as acetosyringone (AS), into the co-cultivation medium of dicotyledonous plant species [9–11], monocotyledonous plant species [12–14], fungi [15,16] and cultured human cell lines [17]. However, one major limitation is that the addition of AS remains ineffective for many plant species, such as legumes [18–21]. Thus, the identification of novel chemicals is still required for improving the transformation efficiency of *Agrobacterium* amongst a broader host range.

*Lotus japonicus* is a diploid legume (2n = 12) with a small genome size (472.1Mb) that reproduces by self-fertilization and has a short generation time. It has been used as a model legume in genetic, genomic and physiological studies, particularly in the field of symbiotic plant-microbe interactions [22,23]. Large scale genomic resources for *L*. *japonicus* have been developed, including whole genome sequences [24], a *LORE1* retrotransposon mutagenesis system, as well as transcriptome, proteome and metabolome profiling [25–31]. The most commonly used accession in the research field is Gifu B-129 which has a high transformation efficiency by *Agrobacterium*[22,32,33], and is best suited for insertional mutagenesis by T-DNA or transposable elements [34,35]. However, Gifu B-129 can be problematic in propagation of subsequent generations because this accession needs high-intensity light irradiation for flowering. Conversely, Miyakojima MG-20, which was used for the whole genome sequencing project [24] can be easily induced to flower under low-intensity light conditions and has a shorter generation cycle by approximately one month [36]. Despite the availability of whole genome sequences, this accession has been rarely used in gene discovery and functional characterization because of its difficulty in transformation.

The aim of this study is to identify novel chemicals that improve the success rate of transgene delivery in plants using the *Agrobacterium*-mediated transformation method. Chemical screening was performed using LATCA (Library of AcTive Compounds on *Arabidopsis*) (http://cutlerlab.blogspot.jp/2008/05/latca.html) and Miyakojima MG-20 as a host plant. LATCA contains a total of 3600 compounds, including 1000, 1600 and 400 compounds that are hypocotyl elongation inhibitors of *Arabidopsis*, growth inhibitors of yeast and herbicides, respectively. A phenolic compound named chloroxynil (CX), was identified from this chemical library that showed a significant improvement of transgene delivery. This finding is expected to contribute to genomic studies in *L*. *japonicus* and several other plant species.

## Materials and Methods

### Chemical Screening

The LATCA chemical library consisted of 3600 compounds as 2.5 mM stock solutions dissolved in DMSO. *L*. *japonicus* accession Miyakojima MG-20 was used as a host plant for transformation. *Agrobacterium* infection was performed as described previously with a few modifications [33,37]. The seeds were sterilized using 1% sodium hypochlorite and grown on germination medium (3.3 g/L B5 salt, 30 g/L sucrose, 0.2 mg/L 6-benzylaminopurine (BA), and 0.2% phytagel (Sigma-Aldrich, USA), pH5.7) at 25°C, 16h light/8h dark cycle for 4 days. *Agrobacterium* strain EHA105 containing pCAMBIA1105.1R (S1 Fig, Cambia, Australia) was grown overnight at 28°C on a rotary shaker in 20 ml of YEP liquid medium supplemented with 10 mg/L streptomycin until OD_600_ = 0.3~0.4. The bacteria cells were collected by centrifugation at 6000 rpm for 10 min at 25°C. The pellet was resuspended in 10 ml of co-cultivation medium (3.3 g/L B5 salt, 30 g/L sucrose, 0.2 mg/L BA, and 0.05 mg/L naphthalene acetic acid (NAA), pH 5.1). Hypocotyls of 4 day-old seedlings were excised in the bacterial suspension and then inoculated for 1h. Nine segments were transferred to a two-layer sterile filter paper soaked with 0.5 ml of co-cultivation medium in a culture dish. Each compound from LATCA was added to each dish at a final concentration of 5 μM. 0.2% DMSO was used as a negative control. The dishes were incubated at 25°C in the dark for 5 days. GUS reporter gene activity was measured as described by Jefferson et al. (1987)[38]. All compounds were analyzed in duplicate.

### *L*. *japonicus* Transient Transformation Assay

The hypocotyls of 4 day-old seedlings were sterilely excised in the suspension of *Agrobacterium strain* EHA105 or GV3101 containing pCAMBIA1105.1R and the segments were incubated in co-cultivation medium with each chemical, CX, AS, bromoxynil (BX) or ioxynil (IX) in the dark at 25°C. After 5 days, GUS reporter gene activity was measured as the method described above. All analyses were carried out in duplicate.

### Plant Growth Measurements

Hypocotyl segments were incubated in co-cultivation medium with different concentrations of CX or AS in the dark at 25°C. After 5 days, the fresh weight per hypocotyl segment was measured. The measurements were performed in duplicate.

### *Agrobacterium vir* Gene Expression Analysis

*A*. *tumefaciens* strain EHA105 cells were grown and resuspended in co-cultivation medium with each chemical by the methods described above and then incubated for 20 h at 25°C. The pellets were collected at 15000 rpm for 10 min at 4°C. Total RNA was isolated from each pellet using Nucleospin RNA (TAKARA Bio Inc., Japan). Reverse transcription was performed using PrimeScript II 1st strand cDNA Synthesis Kit (TAKARA Bio Inc., Japan). Quantitative RT-PCR was carried out using FastStart Universal SYBR Green Master (Rox) (Roche Applied Science, Germany) and ABI7900HT Fast Real-Time PCR System (Life Technologies Corporation, USA). The primer sets of *VirB1* gene were as follows: 5’- GCCCCATCAGTTGCGACATC-3’ and 5’-GACGACTTGGGTTGCTTGGC-3’. The PCR conditions were as follows: 95°C for 10 min for the activation of Taq DNA polymerase, followed by 95°C for 10 sec and 60°C for 30 sec for the cycling stage of 40 cycles, which was followed by melting curve analysis. 16S rRNA was used as an internal control with primers 5’-GCGATGTCGAGCTAATCTCC-3’ and 5’-CGCACTACCTTCGGGTAAAA-3’. All quantifications were made in duplicate on RNA samples obtained from three independent experiments.

### Stable Transformation of *L*. *japonicus*

*Agrobacterium* infection of hypocotyl segments of *L*. *japonicus* treated with 5 μM CX was performed by the method described above. The segments were transferred to shoot induction medium (SIM; 3.3 g/L B5 salt, 30 g/L sucrose, 0.2 mg/L BA, 0.05mg/L NAA, and 0.2% phytagel, pH 5.7) containing 50mg/L hygromycin and 25 mg/L meropenem (Wako Pure Chemicals, Japan) and incubated at 25°C, 16h light/8h dark cycle. Explants were transferred to new SIM containing 12.5 mg/L meropenem every 2 weeks. After six weeks, the explants were transferred to shoot elongation medium (SEM; 3.3 g/L B5 salt, 30 g/L sucrose, 0.2 mg/L BA, and 0.2% phytagel, pH 5.7) containing 12.5 mg/L meropenem and incubated under same conditions. Explants were transferred to new SEM containing 12.5 mg/L meropenem every 2 weeks. After 4 weeks, the elongated shoots were excised and transferred to root induction medium (RIM; 3.3 g/L B5 salt, 30 g/L sucrose, 0.05 mg/L NAA, and 0.2% phytagel, pH 5.7), and incubated for 2 weeks under the same conditions. Rooted explants were transferred to root elongation medium (REM; 3.3 g/L B5 salt, 10 g/L sucrose, and 1% agar (Wako Pure Chemicals, Japan), pH 5.7) for 4 weeks and incubated under same conditions.

To confirm transgenic plants, genomic DNA was extracted from leaves of wild-type and transgenic lines as described previously [39]. Putative transgenic plants were screened by PCR using the forward primer GUSPlusF (5’-TTAACGAAGCGAGCAATGTG -3’) and reverse primer NOStermR (5’-GATCTAGTAACATAGATGACACCGC-3’). PCR was performed under the following conditions: one cycle of 96°C for 2 min, followed by 35 cycles of 96°C for 10 s, 62°C for 15 s, and 72°C for 30 s, with a final 5 min extension at 72°C. Southern blotting analysis was performed on screened transgenic and non-transgenic plants. Twelve micrograms of genomic DNA were digested with *Hind*III (New England Biolabs, Ipswich, MA), which were unique sites in the plasmid. The digested DNA was separated by electrophoresis on a 1% (w/v) agarose gel and transferred to Hybond-N+ nylon membrane (GE Healthcare, NJ, USA). An 1186 bp DNA fragment, to be used as a probe, was amplified by PCR using the template plasmid, pCAMBIA1105.1R and the primers, GUSPlusF and NOStermR (S1 Fig). Probe labelling, hybridization and immunological detection were performed with AlkPhos direct labelling and detection system (GE Healthcare, NJ, USA) according the manufacturer’s instructions. The signal was detected using Hyperfilm ECL (GE Healthcare, NJ, USA).

### Quantitative GUS Assay in Rice

*Agrobacterium*-mediated transformation of *Oryza sativa*, cv. Nipponbare was performed as described previously [40]. Nine calli were transformed by *Agrobacterium* strain EHA105 containing pCAMBIA1105.1R and cultured as described for *L*. *japonicus* using AAM medium containing different concentrations of CX or AS. After 5 days of co-cultivation, calli were used for measuring GUS reporter gene activity as described for *L*. *japonicus*. All analyses were carried out in duplicate.

## Results

### Identification of Compounds Enhancing *Agrobacterium*-Mediated Genetic Transformation

**Fig 1:**
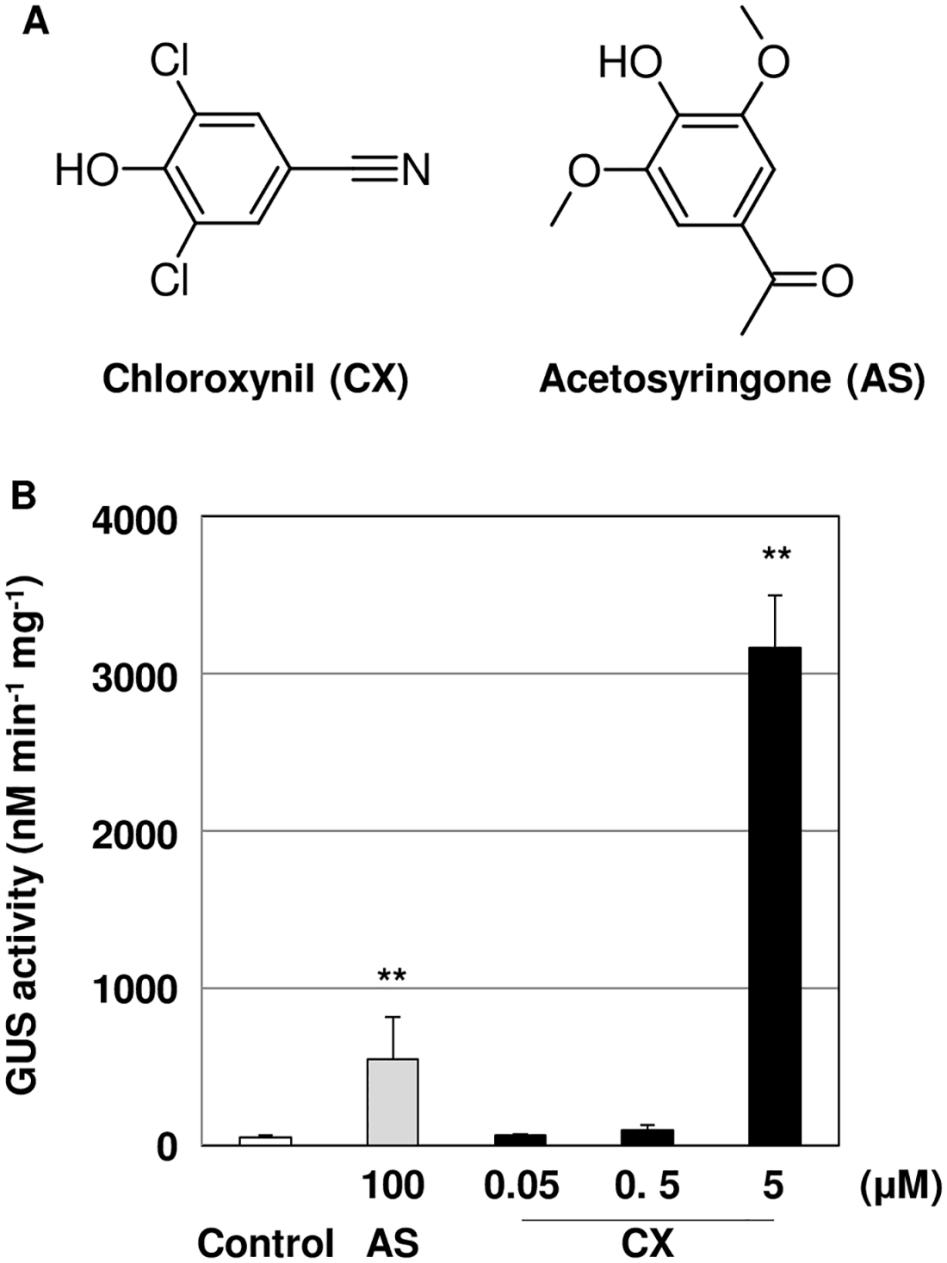
Transformation efficiency of *Agrobacterium* by Chloroxynil (CX) treatment in *L*. *japonicus*. A) The structures of CX and Acetosyringone (AS). (B) Quantitative analysis of GUS activity in *L*. *japonicus*. The hypocotyl segments of 4 day-old seedlings were transformed by *Agrobacterium tumefaciens strain* EHA105 on co-cultivation medium containing different concentrations of CX for 5 days. DMSO (0.2% was used as a negative control. The bars represent the mean ± SE of three replicates (n = 9). Asterisks indicate significant differences between the control and treated segments (**: P<0.01) by Student’s t-test.

We used a chemical library (LATCA) to screen for compounds that improved *Agrobacterium*-mediated genetic transformation. Hypocotyl explants from 4 day-old seedlings were inoculated with *Agrobacterium tumefaciens* strain EHA105 harboring the pCAMBIA1105.1R vector, and co-cultivated with 5 μM of each chemical compound from LATCA. After 5 days, the transient transformation efficiency in hypocotyl explants was detected using a GUS fluorometric assay. As a result of the screen, chloroxynil (CX) was identified as an activator of *Agrobacterium*-mediated transformation (Fig 1A). In order to determine whether CX acts in a dose-dependent manner, we analyzed the effective range of CX concentrations on increasing transformation by measuring GUS reporter activity. Treatment with 5 μM CX had 61.3-fold higher GUS reporter activity than that of control (Fig 1B), and this treatment showed a 5.8-fold higher reporter activity than treatment with 100 μM acetosyringone (AS) (Fig 1A), a known inducer of *virulence* (*vir*) genes in *A*. *tumefaciens* (Fig 1B)[41]. Both 0.05 μM and 0.5 μM of CX treatments showed no significant GUS reporter activity. GUS activity of 50 μM CX-treated samples was not analyzed due to the toxic effect of CX (data not shown).

### Herbicidal Effect of CX on Plant Growth

**Fig 2:**
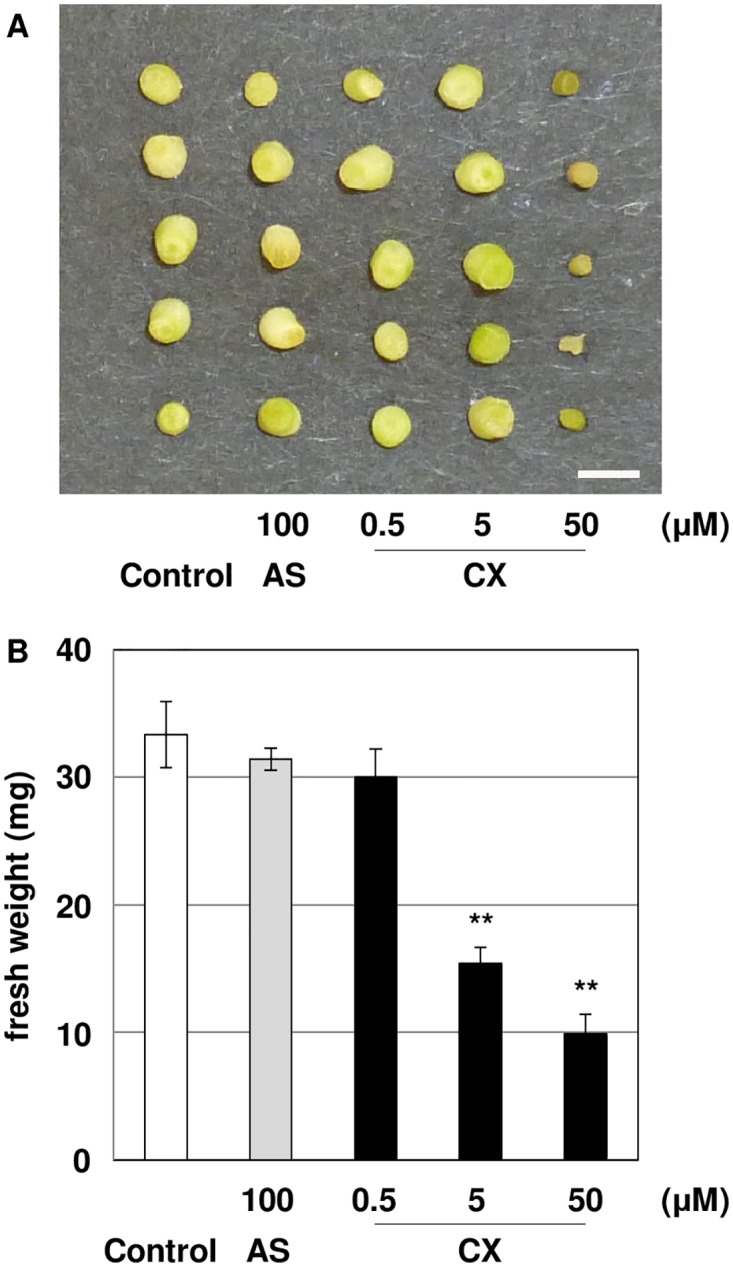
Growth inhibition of *L*. *japonicus* treated with CX. (A) Hypocotyl segments were incubated on co-cultivation medium containing different concentrations of CX for 5 days. (B) Fresh weight of hypocotyl segments treated with CX for 5 days. The segments were weighed in triplicate. The bars represent the mean ± SE of three replicates. Bar represents 1 cm. Asterisks indicate significant differences between the control and treated segments (**: P<0.01) by Student’s t-test.

Benzonitrile herbicides, which include CX and bromoxynil (BX), have been known to induce rapid cell death in *Arabidopsis thaliana*[42]. Thus, the degree of CX-mediated growth inhibition was investigated in *L*. *japonicus*. Hypocotyl explants suffered serious damage with 50 μM CX treatment (Fig 2A), and the fresh weight per explant was significantly less than explants treated with 5 μM CX (Fig 2B). No significant differences were observed in the appearance of explants and fresh weight in 0.5 μM CX and 100 μM AS treatment (Fig 2). Based on these results, it was concluded that the growth inhibition in *L*. *japonicus* occurred at concentrations above 5 μM of CX treatment.

### Induction of *Agrobacterium vir* Gene Expression by CX Treatment

**Fig 3:**
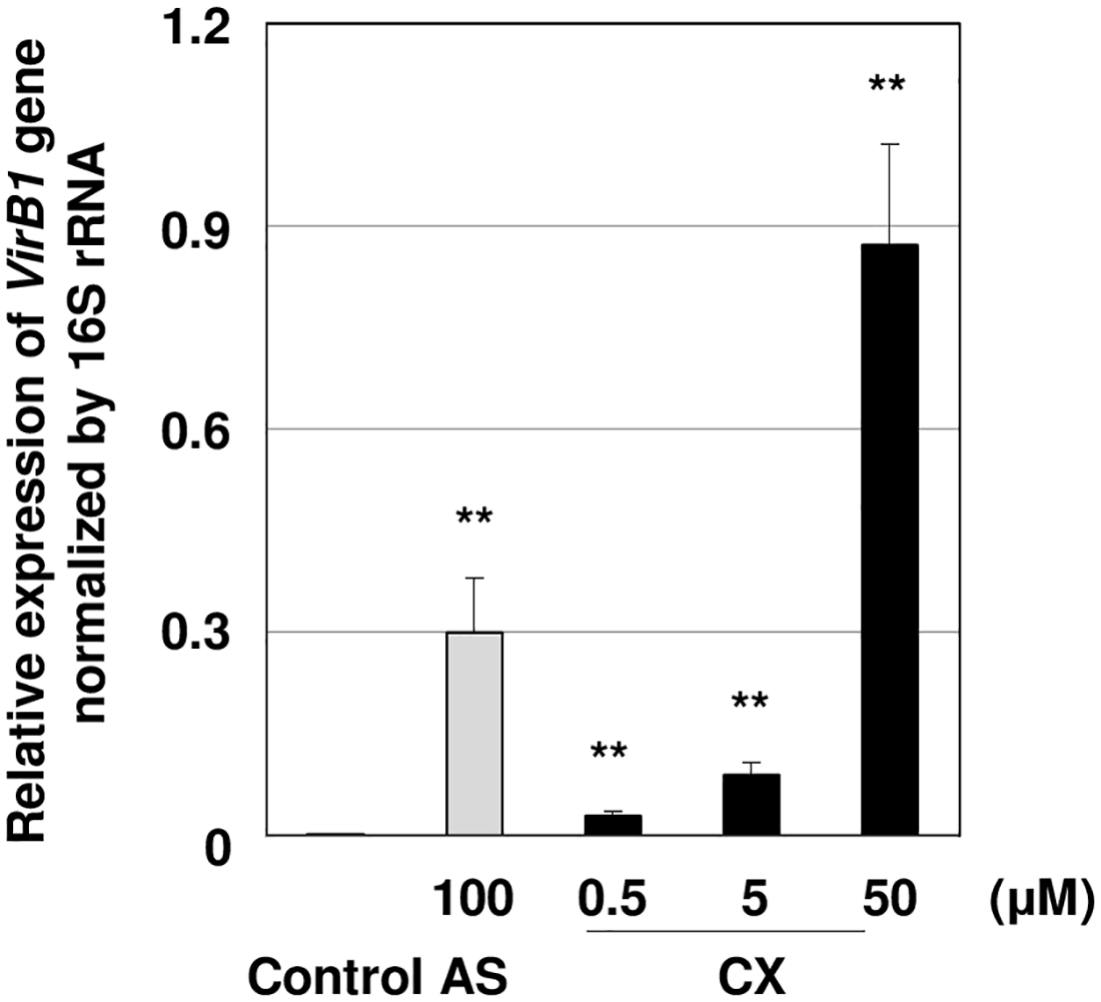
*VirB1* expression treated with CX in *Agrobacterium tumefaciens* strain EHA105. *Agrobacterium* grown on liquid YEP medium (OD_600_ = 0.1) was cultivated in co-cultivation medium containing different concentrations of CX at 25°C for 20 h. The bars represent the mean ± SE of three independent RNA isolations. The relative expression level is normalized by 16S rRNA as an internal control. Asterisks indicate significant differences between the control and each treatment (**: P<0.01) by Student’s t-test.

To determine whether *Agrobacterium vir* genes are induced by CX treatment, *vir* gene expression level was quantified using real-time qPCR. The *VirB1* gene was activated by CX treatment in a dose-dependent manner (Fig 3) and gene induction was highest when treated with 50 μM CX (Fig 3).

### Effect of CX Treatment on *L*. *japonicus* Transformation by Different *Agrobacterium* Strain

**Fig 4:**
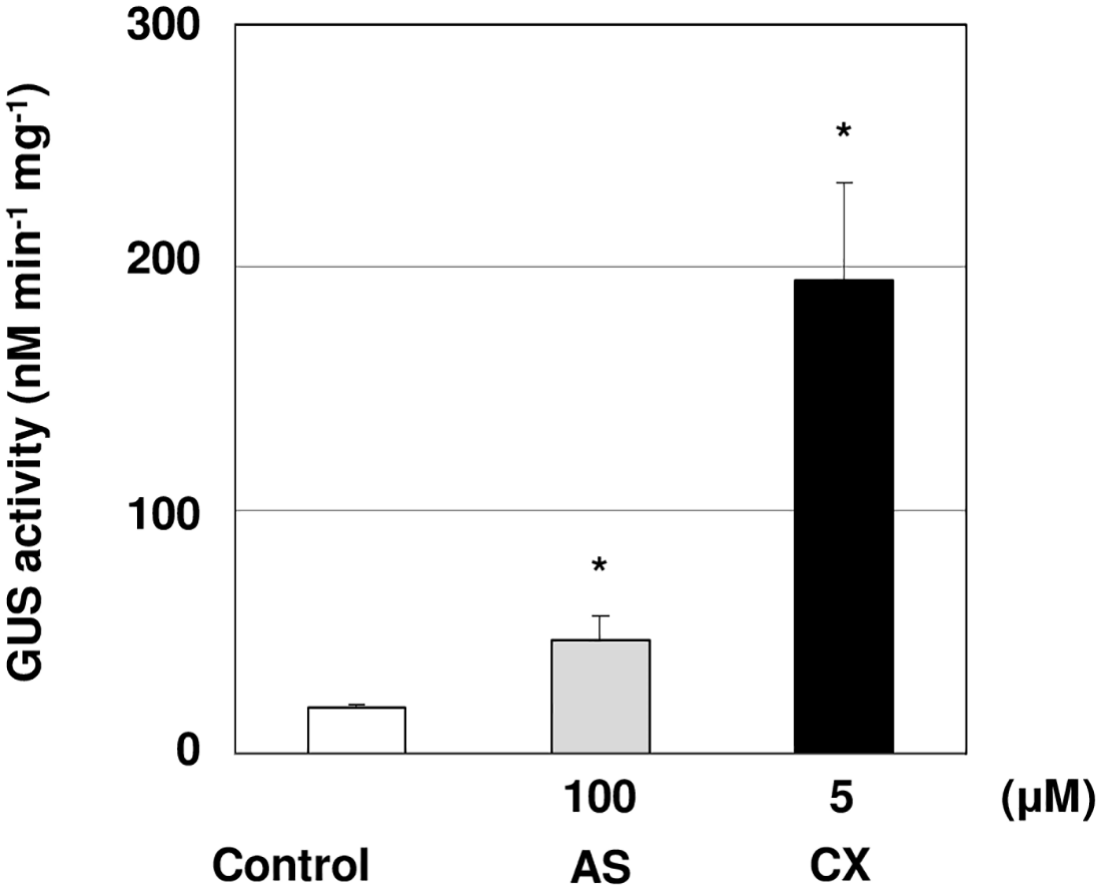
Transformation efficiency for *Agrobacterium* by different *Agrobacterium* strains. The hypocotyl segments of 4 day-old seedlings were transformed by *Agrobacterium tumefaciens strain* GV3101 on co-cultivation medium containing 5 μM CX for 5 days. The bars represent the mean ± SE of three replicates (n = 9). Asterisks indicate significant differences between the control and treated segments (*: P<0.05) by Student’s t-test.

To investigate the effect of CX treatment on *L*. *japonicus* transformation in different *Agrobacterium* strains, hypocotyl explants of *L*. *japonicus* MG-20 were co-cultivated with *Agrobacterium tumefaciens* strain GV3101 harboring the pCAMBIA1105.1R plasmid and treated with 5 μM CX. GUS activity of plants treated with CX was 10.2-fold higher than that of the control (Fig 4). These results showed that CX improved *L*. *japonicus* transient transformation using two different *Agrobacterium* strains.

### Effect of CX Treatment on Rice Transformation

**Fig 5:**
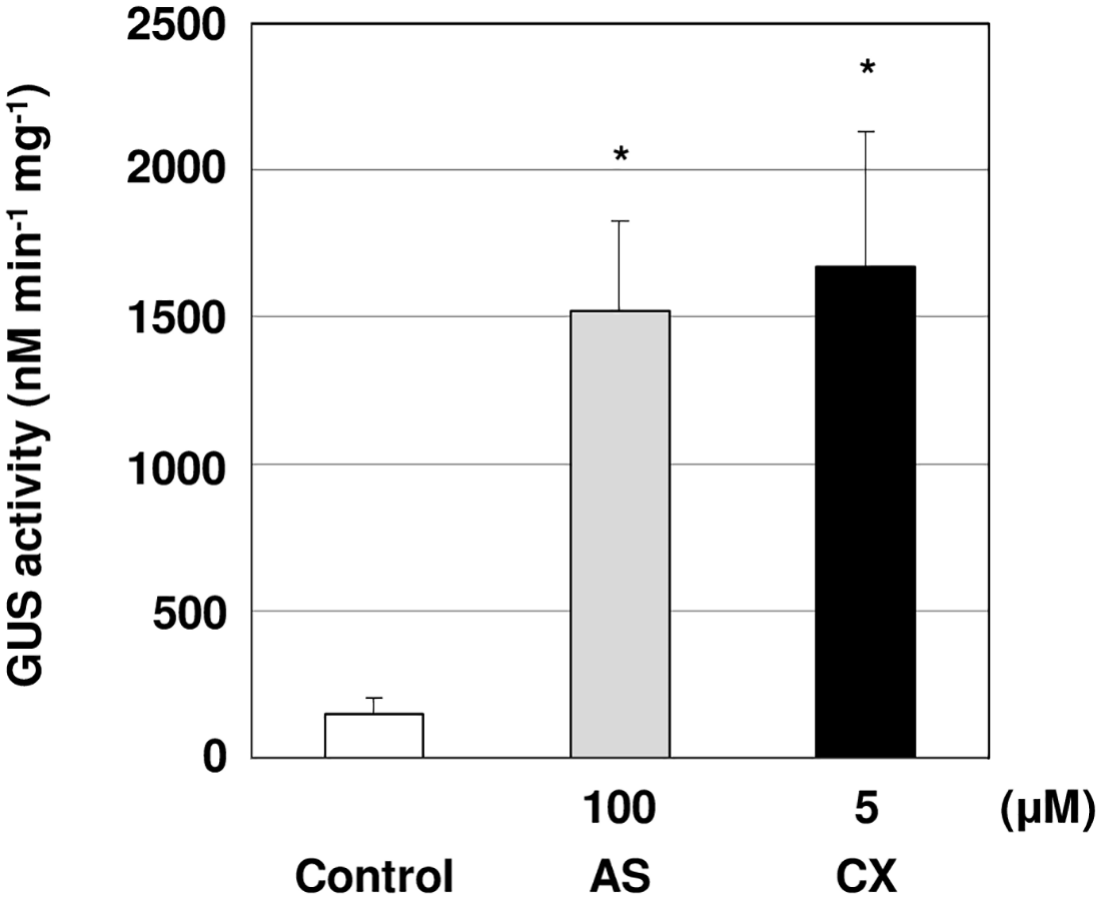
Transformation efficiency of *Agrobacterium* by CX treatment in rice. (A) Quantitative analysis of GUS activity in rice. The calli were co-cultivated using the same method as *L*. *japonicus*. The bars represent the means ± SE of three replicates (n = 5). Asterisks indicate significant differences between the control and treated calli (*: P<0.05) by Student’s t-test.

To evaluate the effect of CX treatment in the genetic transformation of monocots, rice transformation was performed using CX. As a result, GUS activity of 5 μM CX treated rice calli was 10.6-fold higher than that of control (Fig 5). These results suggested that CX treatment can enhance the transformation efficiency of monocotyledonous species as well as dicotyledonous species.

### Effect of the CX Treatment on Transformation Efficiency

**Fig 6:**
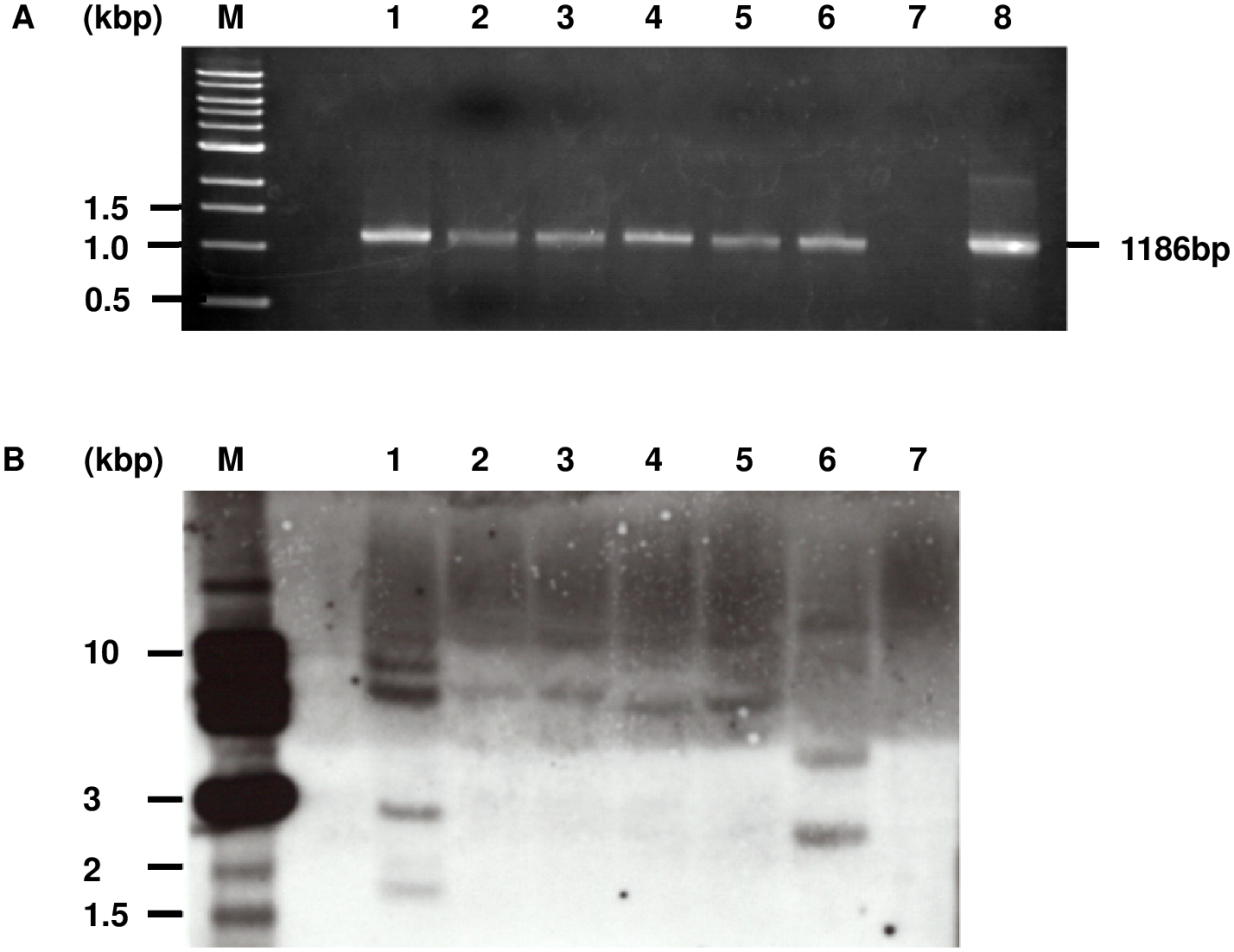
PCR analysis and southern blot analysis of the transgenic lines in *L*. *japonicus*. (A) PCR detection of the transgene in transgenic lines. M: 1kb ladder marker, lane 1: transgenic plant of control, lane 2–3: transgenic plantsregenerated with AS treatment, lane 4–6: transgenic plant regenerated with CX treatment, lane 7: non-transformed *L*. *japonicus* (negative control), lane 8: pCAMBIA1105.1R (positive control). (B) Southern blot analysis of transgene in transgenic lines. The *Hind*III-digested genomic DNA was hybridized with *GUSPlus* probe. M: 1kb ladder marker, lane 1: transgenic plant of control, lane 2–3: transgenic plant regenerated with AS treatment, lane 4–6: transgenic plant regenerated with CX treatment, lane 7: non-transformed *L*. *japonicus* (negative control).

The effect of the CX treatment on transformation efficiency was verified by two independent experiments (Table 1). The average callus induction efficiency with 5 μM CX treatment (50.2%) was approximately 1.8 and 3.3-fold higher than that of the control (27.4%) and AS treatment (15.0%), respectively. A total of 15 hygromycin-resistant plants were regenerated from the calli induced by 5 μM CX treatment. Three of the 15 plants were confirmed positive by PCR for the transgene (Fig 6A and Table 1). To confirm the gene integration and copy number of the transgene in transgenic plants, genomic DNA from all positive transgenic lines were analyzed by Southern blot. The copy number of the transgene in the lines regenerated by CX treatment was not significantly different that of the lines regenerated by control and AS treatment (Fig 6B). No hybridization of the labeled probe was detected in wild-type plants (Fig 6B).

**Table 1:** Effect of 100 μM AS and 5 μM CX treatment on callus induction and transformation efficiency in L. japonicas accession MG-20.

Experimet		segments	Hygromycin-resistant calli^a^	Callus induction rate (%)^b^	Hygromycin-resistant plants	PCR-positive plants	Transformation efficiency (%)^c^
1	control	100	28	28.0	6	0	0.0
	AS	75	14	18.7	3	0	0.0
	CX	100	45	45.0	3	0	0.0
2	control	225	61	27.1	10	1	0.4
	AS	225	31	13.8	12	2	0.9
	CX	225	118	52.4	12	3	1.3
Total	control	325	89	27.4	16	1	0.3
	AS	300	45	15.0	15	2	0.7
	CX	325	163	50.2	15	3	0.9

^a^ Number of calli resistant to 50mg/L hygromycin

^b^ (Number of hygromycin resistant calli / Number of segments) X 100

^c^ (Number of PCR-positive plants / Number of segments) X 100

### Influence of CX Analogues on *L*. *japonicus* Transformation

*Agrobacterium*-mediated transformation efficiency was investigated using two CX analogues, BX and ioxynil (IX) (S2A Fig). As a result, GUS activity with 5 μM BX treatment was 18.1-fold higher than that of control (S2B Fig). Both 0.05 and 0.5 μM BX treatments did not show any significant improvement of transformation efficiency relative to the control, whereas GUS activity of 50 μM BX-treated samples were not analyzed due to severe growth inhibition (data not shown). IX treatment did not show any significant effect on the reporter activity at any concentration (S2B Fig). The fresh weight per explant was reduced in both BX and IX treatments similar to CX (S3 Fig). The *Agrobacterium VirB1* gene was activated by BX treatment, but not by IX treatment (S4 Fig).

## Discussion

In this study, we identified CX as an activator of *Agrobacterium*-mediated transformation. A previous study reported that phenolic compounds and their analogues induce *vir* genes in *Agrobacterium*[43]. Although *VirB1* gene expression levels in 5 μM CX treated explants were lower than that those treated with 100 μM AS (Fig 3), transient transformation efficiency was higher in 5 μM CX-treated samples (Fig 1B), suggesting that the mechanism is not simply correlated with the level of *VirB1* gene expression and could indicate that there are optimal *VirB1* levels below the levels induced by AS and /or that the mechanism involves multiple sites of action. Our results indicated that 5 μM CX caused growth inhibition but 100 μM AS did not (Fig 3). It has been reported that several herbicides, such as 2,4-D and glyphosate, impair the defense responses to fungi [44,45]. Though the interaction between *Agrobacterium*-mediated gene transfer and the herbicidal efficacy of CX is unknown, we predict that cellular damage by CX treatment may contribute to enhancements in gene transfer efficiency.

Under the 50 μM CX treatment, *VirB1* gene induction was remarkably increased (Fig 3). However this condition is toxic and causes severe damage to the cells in *L*. *japonicus* (Fig 2). CX is a benzonitrile herbicide and its analogues, BX and IX, bind the D1 protein in photosystem II and inhibit electron transport, which induces degradation of the D1 protein [46]. Moreover, previous studies reported that BX treatment inhibits the electrogenic proton pump by cytosol acidification [47,48]. Therefore, it is likely that that CX acts in a similar herbicidal mechanism to BX and IX. Meanwhile, our results indicated that IX did not affect transformation efficiency. Therefore we concluded that that the herbicidal targets of these compounds were not the primary cause of the increase in transformation observed.

Since *Agrobacterium* VirA protein senses phenolic compounds such as AS [49] and regulates expression of *vir* genes, it is possible that the VirA protein also senses CX. Although *VirB1* gene induction in BX and IX treatments were lower than that of CX treatment (S4 Fig), the growth inhibition was similar between all three chemical treatments (S3 Fig). Therefore, we propose that the lower transformation efficiency in BX and IX treatments are due to lower *VirA* sensing ability caused by structural differences.

Three independent transgenic lines were established by CX treatment. (Fig 6 and Table 1). It is worth mentioning that CX did not seem to have a negative effect on callus induction or regeneration process. CX treatment can improve transient transformation using multiple *Agrobacterium* strains and host plant species (Figs 4 and 5). Consequently, it is possible that CX treatment may improve efficient *Agrobacterium*-mediated transformation of various plant species.

## Supporting Information

S1 Fig Restriction digest map of pCAMBIA1105.1R. Plasmid map was generated using the SnapGene Viewer (GSL Biotech LLC). (TIF)

S2 Fig Transformation efficiency for *Agrobacterium* by BX and IX treatment in *L*. *japonicus*. (TIF)

S3 Fig Growth inhibition of *L*. *japonicus* treated with 5 μM BX and IX. (TIF)

S4 Fig *VirB1* expression treated with 5 μM BX and IX in *Agrobacterium tumefaciens strain* EHA105. (TIF)
