# Supplementary figures and images for: A Novel Phenolic Compound, Chloroxynil, Improves Agrobacterium-Mediated Transient Transformation in Lotus japonicus
Source: PLoS One. 2015 Jul 15;10(7):e0131626. doi: 10.1371/journal.pone.0131626 (PMC4503419; doi:10.1371/journal.pone.0131626)

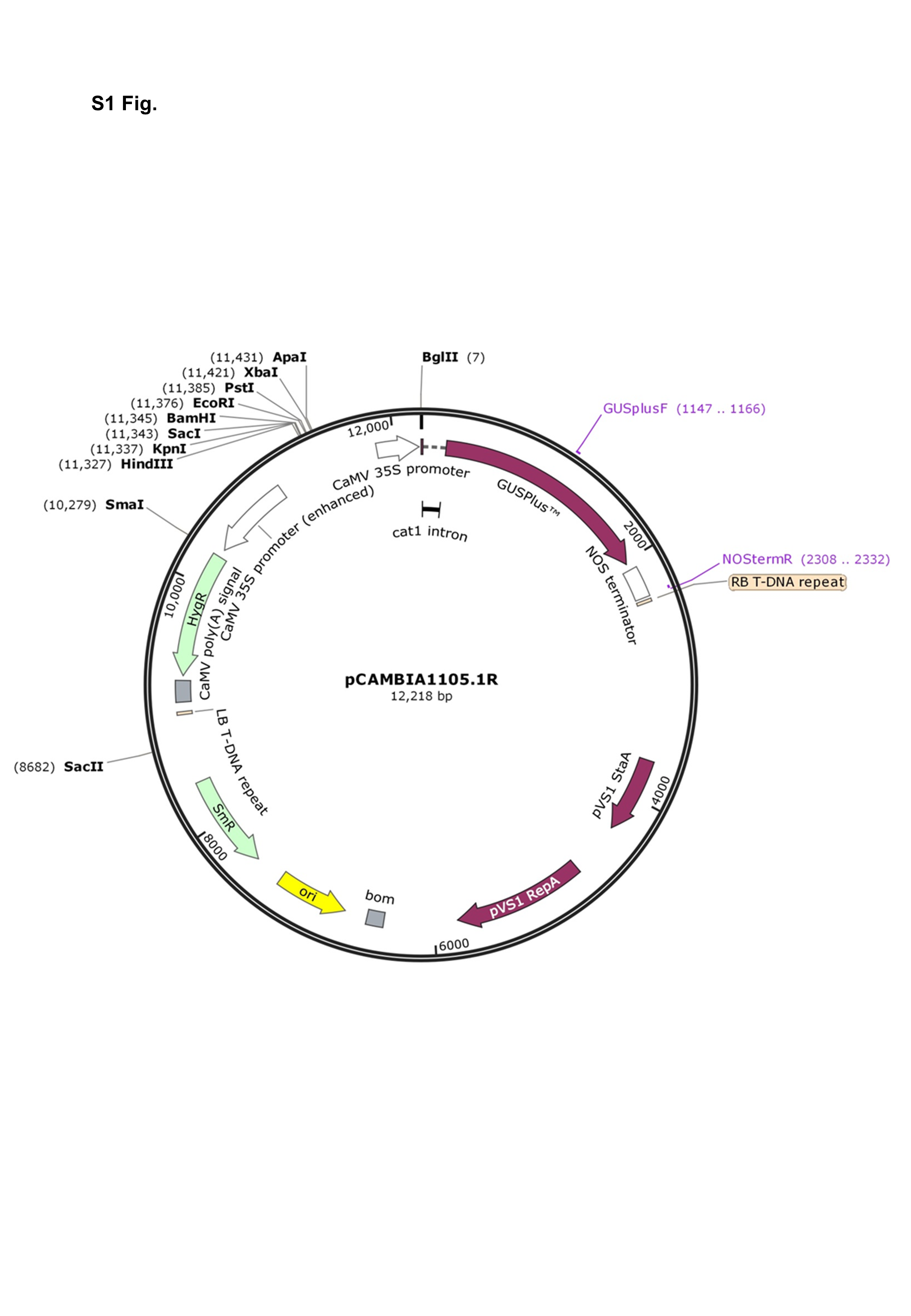

Supplement: S1 Fig — (TIF) [file pone.0131626.s001.TIF]

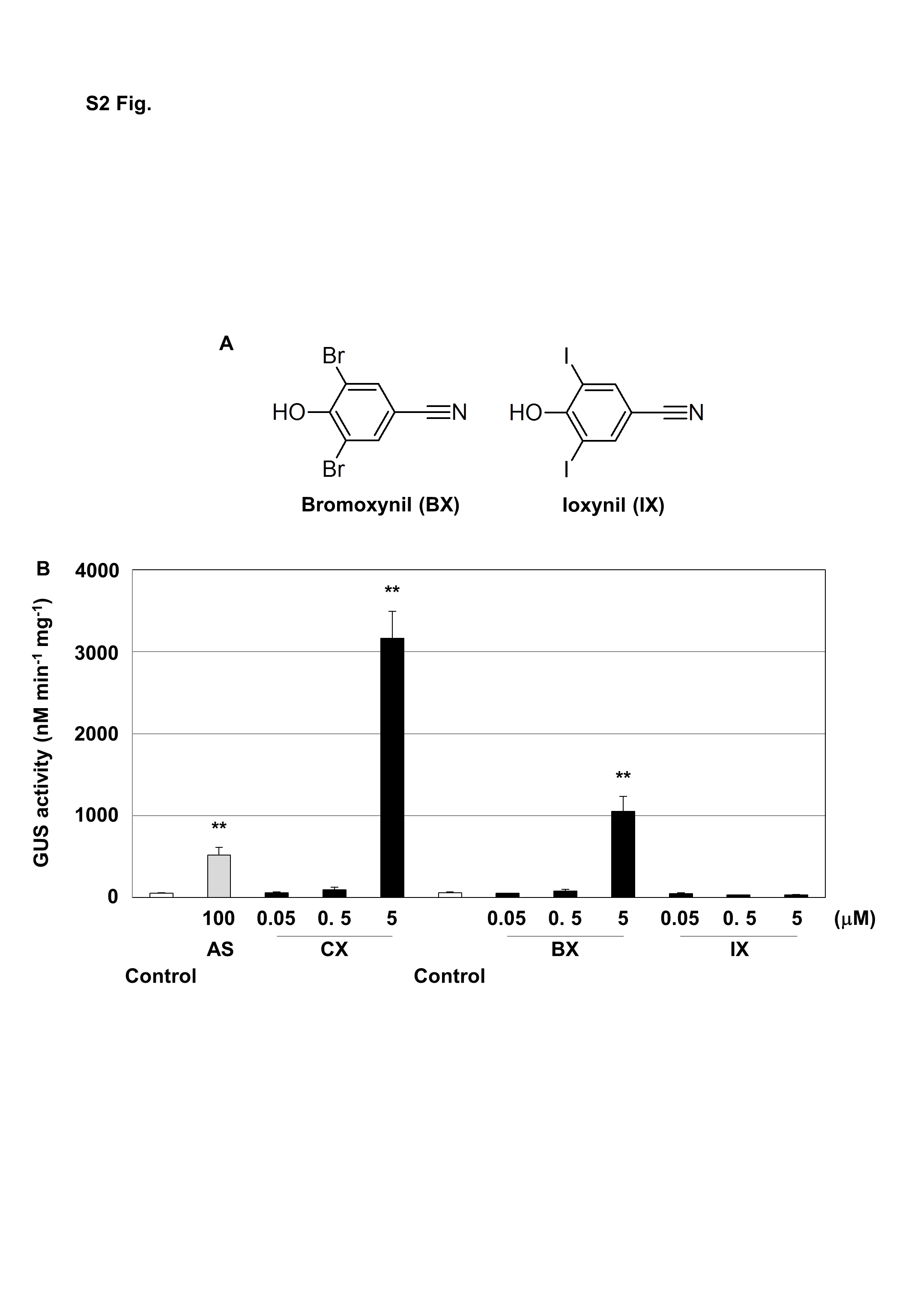

Supplement: S2 Fig — (TIF) [file pone.0131626.s002.TIF]

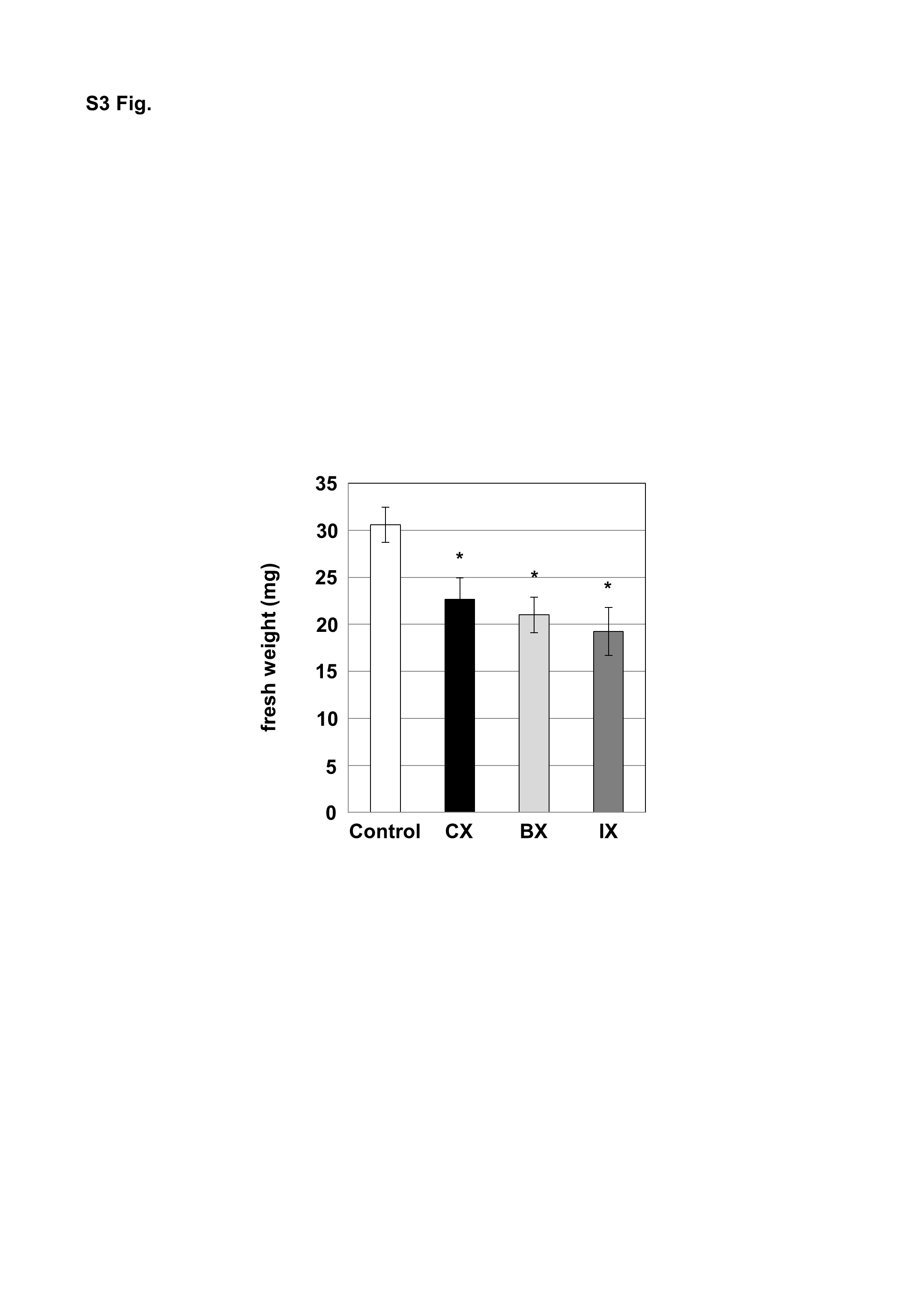

Supplement: S3 Fig — (TIF) [file pone.0131626.s003.TIF]

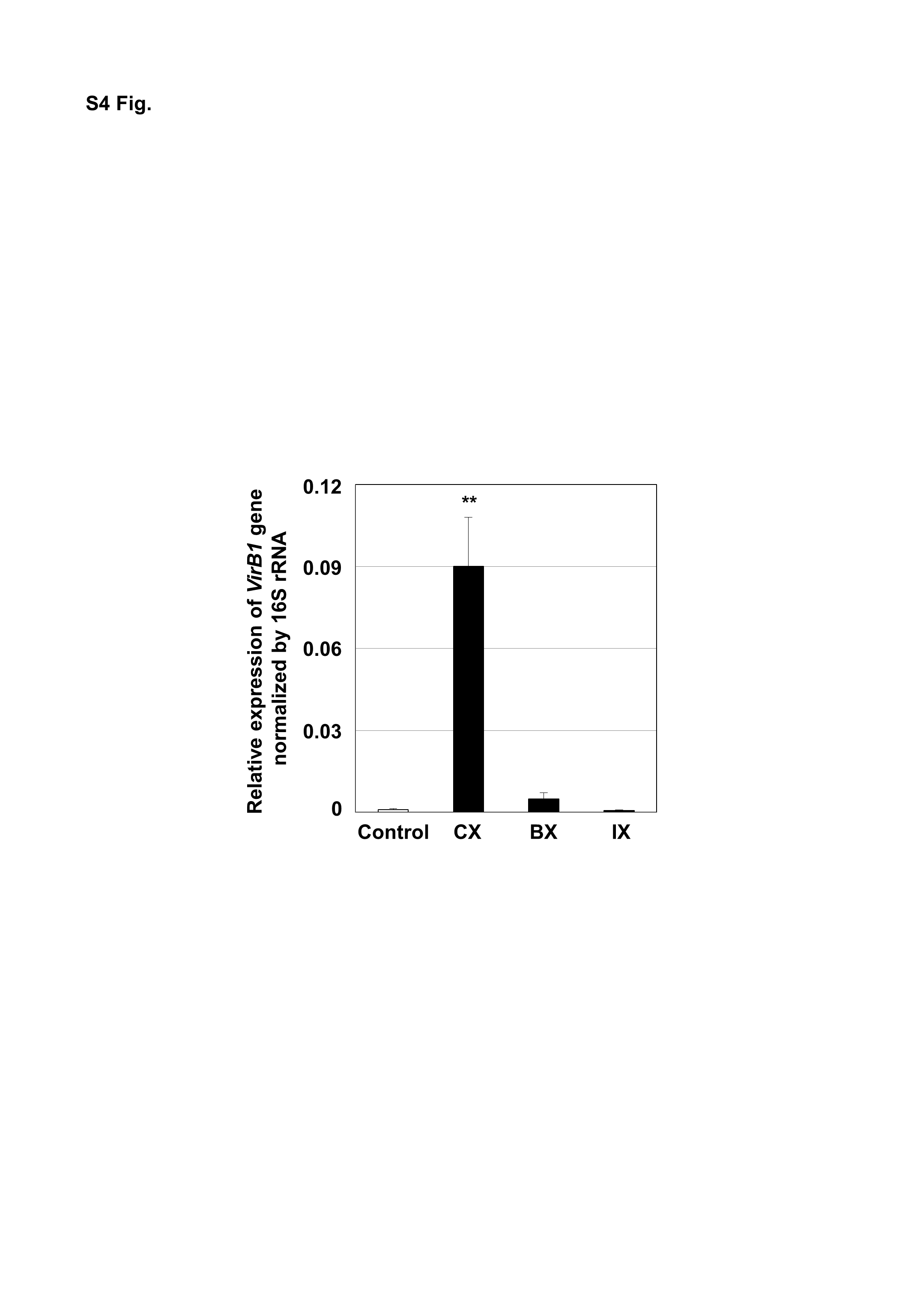

Supplement: S4 Fig — (TIF) [file pone.0131626.s004.TIF]
